# RNA-Seq Analysis of Colorectal Tumor-Infiltrating Myeloid-Derived Suppressor Cell Subsets Revealed Gene Signatures of Poor Prognosis

**DOI:** 10.3389/fonc.2020.604906

**Published:** 2020-11-10

**Authors:** Reem Saleh, Varun Sasidharan Nair, Mahmood Al-Dhaheri, Mahwish Khawar, Mohamed Abu Nada, Nehad M. Alajez, Eyad Elkord

**Affiliations:** ^1^ Cancer Research Center, Qatar Biomedical Research Institute (QBRI), Hamad Bin Khalifa University (HBKU), Qatar Foundation (QF), Doha, Qatar; ^2^ Department of Surgery, Hamad Medical Corporation, Doha, Qatar; ^3^ Biomedical Research Center, School of Science, Engineering and Environment, University of Salford, Manchester, United Kingdom

**Keywords:** myeloid-derived suppressor cells, colorectal cancer, transcriptomic profiling, metabolism, signaling pathways, immune responses

## Abstract

Elevated levels of myeloid-derived suppressor cells (MDSCs), including polymorphonuclear MDSCs (PMN-MDSCs) and immature MDSCs (I-MDSCs), are usually associated with disease progression in cancer patients, including colorectal cancer (CRC). However, biological mechanisms and molecular pathways regulated by MDSC subpopulations in the CRC tumor microenvironment (TME) have not been fully investigated. In this study, we performed transcriptomic analysis of tumor-infiltrating I-MDSCs and PMN-MDSCs isolated from tumor tissues of six CRC patients, compared to antigen-presenting cells (APCs). We also compared the transcriptomic profiles of tumor-infiltrating PMN-MDSCs to I-MDSCs. Our results showed different molecular pathways regulated by each MDSC subset, potentially reflecting their phenotypical/molecular/functional characteristics in the CRC TME. Moreover, we identified gene signatures in PMN-MDSC and I-MDSC of poor overall survival (OS) and disease-free survival (DFS) using the Cancer Genome Atlas (TCGA) dataset from patients with colon adenocarcinoma (COAD). However, functional studies are required to validate these findings.

## Introduction

Myeloid-derived suppressor cells (MDSCs) are a heterogeneous population of myeloid cells, halted at varying stages of maturation/differentiation and exert immunosuppressive activity on other immune cells (1, 2). MDSCs have been divided into different subpopulations based on their phenotypical and functional characteristics; early-stage or immature MDSCs (e-MDSC/I-MDSC) identified as CD33^+^HLA-DR^-/low^CD14^-^CD15^-^, monocytic MDSCs (M-MDSCs) identified as CD33^+^HLA-DR^-/low^CD14^+^CD15^-^, and polymorphonuclear MDSCs (PMN-MDSCs) identified as CD33^+^HLA-DR^-/low^CD14^-^CD15^+^ (1, 3, 4). Myelopoiesis is disrupted in inflammation-related cancers (5), such as colorectal cancer (CRC), leading to increased number of MDSCs in the circulation and tumor tissues (1, 2).

The contribution of MDSCs to cancer pathogenesis and progression is well-established (3, 6). Increased level of MDSCs has been associated with poor prognosis and short survival periods in CRC patients (7–9). MDSCs within the tumor microenvironment (TME) exert their suppressive activity on T cells to inhibit their anti-tumor activities (6, 10). MDSCs mediate immunosuppression by expressing co-inhibitory ligands, such as PD-L1, which induces T cell dysfunction upon the interaction with its receptor PD-1, and by expressing suppressive molecules, such as arginase-1 (ARG1), inducible nitric oxide synthase (iNOS), interleukin-10 (IL-10), and transforming growth factor-β (TGF-β) (11, 12). Furthermore, MDSCs promote tumorigenesis *via* other means, such as the induction of angiogenesis and tumor growth/metastasis, and activation of cancer-associated fibroblasts (CAFs) (12, 13). Up to date, the biological mechanisms and signaling pathways regulated by MDSC subpopulations have not been fully explored. Thus, further insights into these mechanisms and pathways could result in the identification of potential therapeutic targets for cancer.

Previously, we reported increased number of PMN-MDSCs and I-MDSCs in CRC tumor tissues, compared to paired-normal tissues (14), implicating the importance of MDSC role in CRC tumorigenesis and immunosuppression (7). Additionally, we reported the transcriptomic profiles of CRC tumor-infiltrating I-MDSCs and PMN-MDSCs from tumor tissues of only two patients, compared to antigen-presenting cells (APCs) (14). In this study, we extended our investigation and included more CRC patients.

We found that immune response-mediated pathways associated with dendritic cell (DC) maturation (15), triggering receptor expressed on myeloid cells 1 (TREM1) signaling (16), nuclear factor of activated T cells (NFAT)-mediated regulation of immune response (17), and Fcγ receptor-mediated phagocytosis (18) were commonly downregulated in PMN-MDSCs and I-MDSCs, compared to APCs. We also compared the transcriptomic profiles of PMN-MDSCs vs. I-MDSCs and found that pathways supporting tumor growth and survival, related to metabolism, lipid biosynthesis, stress response, increased production of glucose and interaction between DCs and natural killer (NK) cells were different in tumor-infiltrating PMN-MDSCs and I-MDSCs. Therefore, these results may reflect the molecular profile and functional characteristics of each MDSC subset in the CRC TME. We also validated RNA-Seq data by confirming the mRNA expression of selected genes in the different myeloid subpopulations using qRT-PCR. We also utilized the Cancer Genome Atlas (TCGA) from patients with colon adenocarcinoma (COAD) to analyze our RNA-Seq data and identify gene signatures for PMN-MDSC and I-MDSC to predict overall survival (OS) and disease-free survival (DFS).

## Materials and Methods

### Sample Collection and Storage

Tumor tissues (TT) were obtained from six CRC patients (#05, 07, 08, 09, 44, and 53) who underwent surgery at Hamad Medical Corporation, Doha, Qatar. Demographical details and clinicopathological features of study population are shown in **Table 1**. These patients were treatment-naïve prior to surgery and provided written informed consent prior to sample collection. This study was performed under ethical approvals from Hamad Medical Corporation, Doha, Qatar (protocol no. MRC-02-18-012) and Qatar Biomedical Research Institute, Doha, Qatar (protocol no. 2018-018). Tissue specimens were frozen in 1 ml of freezing medium (10% dimethyl sulphoxide (DMSO; Sigma-Aldrich, Missouri, USA), 50% fetal calf serum (FCS; HyClone, GE Healthcare Life Sciences, Utah, USA), and 40% RPMI-1640 medium (Life Technologies, New York, USA)), then stored in liquid nitrogen to be used in batches for subsequent analyses. Tissue specimens were thawed and processed, as previously described, followed by cell staining/sorting (14, 19–21).

**Table 1:** Characteristic features of study populations.

	CRC patients
**Number**	6
**Age**	51 (37-65)^†^
**Gender**	All males
**TNM stage**	
II	2 (CRC #07 & 44)
III	2 (CRC#05 & 08)
IV	2 (CRC #09 & 53)
**Anatomical location**	
Cecum	2 (CRC #07 & 44)
Transverse colon	2 (CRC #08 & 53)
Sigmoid	1 (CRC #09)
Rectum	1 (CRC #05)
**Histological grade**	
G2—Moderately differentiated	All samples

CRC, colorectal cancer.

^†^Data shown represent median (range).

**Figure 1:**
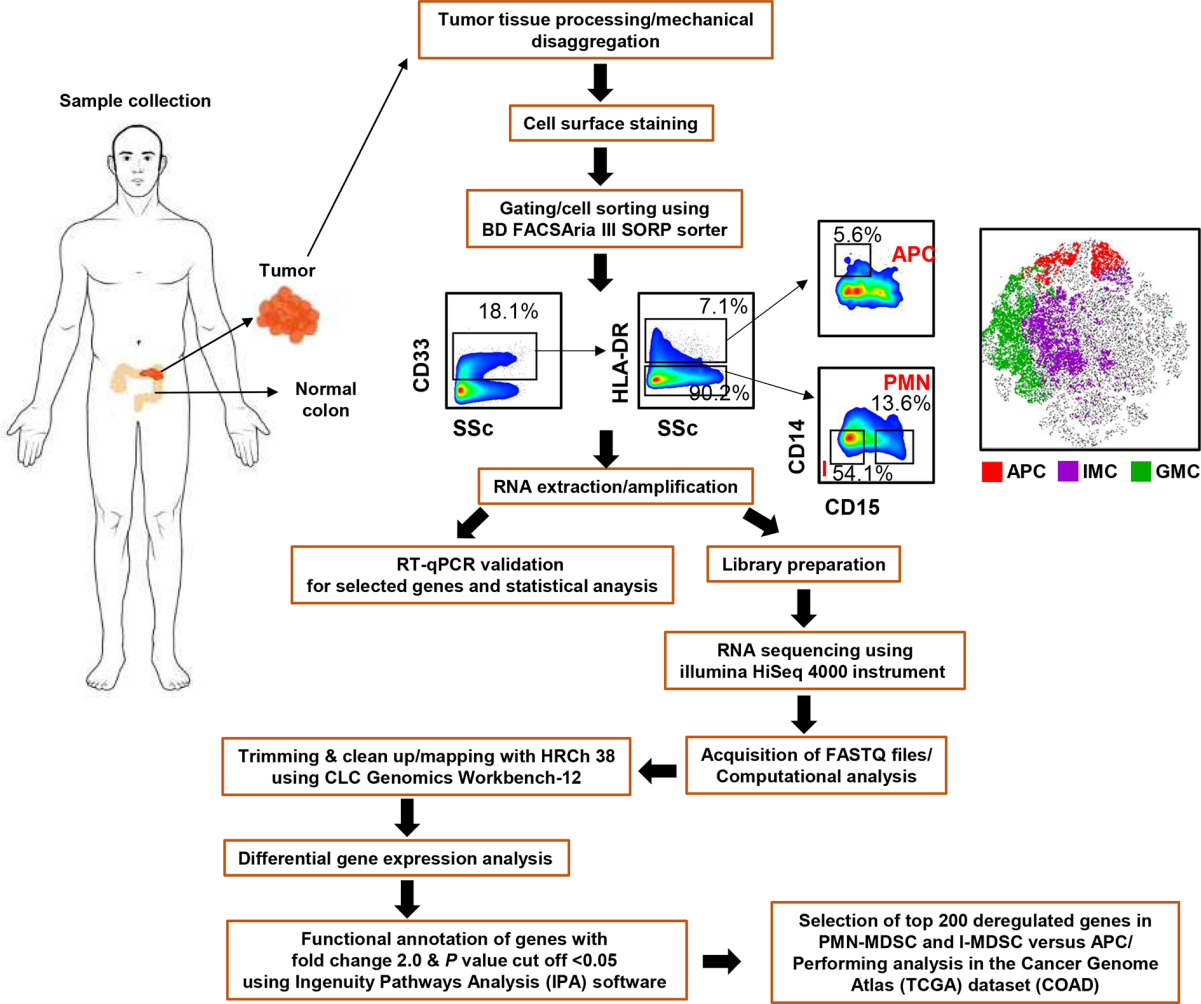
Pipeline for the experimental design and RNA-Seq analysis. Flow chart showing the study design, gating strategy used for cell sorting and bioinformatic tools used for RNA-Seq data analysis.

The flow chart for the experimental design and tissue processing is shown in **Figure 1**. All experiments were performed in accordance with relevant guidelines and regulations.

### Dissociation of Tissue and Cell Sorting

Tissue specimens were thawed and processed, as previously described (14) and as shown in **Figure 1**. Single cell suspensions were isolated from six frozen CRC tissues (Patient #05, 07, 08, 09, 44, and 53) using gentleMACS Dissociator (Miltenyi Biotec), washed and stained with 7-AAD viability dye (eBioscience, San Diego, USA) to gate live cells, and for different cell surface markers; CD33-Fluorescein isothiocyanate (clone HIM3-4; BD Biosciences, Oxford, UK), CD14-phycoerythrin-Cy7 (clone M5E2; BD Biosciences), CD15-allophycocyanin (clone HI98; BioLegend, San Diego, USA), HLA DR-phycoerythrin (clone G46-6; BD Biosciences), to sort APCs and different MDSC subsets, as previously described (14) (**Figure 1**). For cell sorting, BD FACSAria III SORP cell sorter was utilized, with BD FACSDiva software (BD Biosciences). We used stringent gating strategy and applicable measures were taken to ensure minimal sorter-induced cell stress (SICS). High purities of the sorted myeloid cell subpopulations were always checked and confirmed. FlowJo V10 software (FlowJo, Ashland, USA) was used for data analyses.

### RNA Extraction and Reverse Transcription

Total RNA was extracted from sorted pure myeloid subsets, CD33^+^HLA-DR^+^CD14^+^CD15^-^ (APCs), CD33^+^HLA-DR^-^CD14^-^CD15^+^ (PMN-MDSCs), and CD33^+^HLA-DR^-^CD14^-^CD15^-^ (I-MDSCs) using RNAqueous-Micro Total RNA isolation Kit (Thermo Fisher Scientific). Purified RNA was then amplified using 5X MessageAmp II aRNA Amplification Kit (Thermo Fisher Scientific). Before and after amplification, RNA concentrations were determined by Qubit RNA HS and Broad Range Assay Kits, (Invitrogen). For reverse transcription, QuantiTect Reverse Transcription Kit (Qiagen, Hilden, Germany) was used to convert 1 µg of RNA into cDNA.

### Library Preparation

cDNA libraries were generated using Exome TruSeq Stranded mRNA Library Prep Kit (illumina, San Diego, USA) following the manufacturer’s protocol, as previously described (22). Libraries that passed quality control were subjected to clustering using TruSeq PE Cluster Kit v3-cBot-HS (illumina). Sequencing of clustered samples was performed on an illumina HiSeq 4000 instrument using HiSeq 3000/4000 SBS kit (illumina).

### RNA Sequencing Data and Functional Annotation Analyses

As previously described, 150 bp depth paired-ends reads were trimmed and aligned to the hg19 human reference genome in CLC Genomics Workbench-12 (Qiagen) using default settings (14, 22). The abundance of gene expression is determined by the score of TPM (Transcripts Per Million), mapped reads in CLC Genomics Workbench 12. Hierarchical clustering, principal component analysis (PCA) and differential gene expression analyses were performed, as previously described (23), using 2.0-fold change and *P* value <0.05 cutoffs. Ingenuity Pathways Analysis (IPA) software (Ingenuity Systems; www.ingenuity.com) was utilized to perform functional and pathway analyses on differentially expressed genes, as described previously (14, 22). Raw data comparing myeloid subsets are shown in **Supplementary Table 1**. The flow chart for the bioinformatic tools used for RNA-Seq analysis is shown in **Figure 1**. Protein-protein interaction (PPI) networks among the significantly up/downregulated genes were determined by web-based online tool, STRING V11.0 (http://string-db.org).

### Quantitative Real-Time Reverse Transcription PCR

QuantStudio 6/7 Flex Real-time PCR system (Applied Biosystems, California, USA) was used to perform qRT-PCR for genes including CSF2, IL1B, PRF1, GZMA, IFNG, IL2RA, CD40, and β-actin with PowerUp SYBR Green Master Mix (Applied Biosystems). The relative mRNA expression was determined by the normalization to β-actin, and represented as the mean (log_10_) ± standard error of the mean (SEM). **Supplementary Table 2** lists the sequences for the primers used.

### The Cancer Genome Atlas Analysis for RNA Sequencing Data

From our RNA-Seq data, the top 200 upregulated genes and 200 downregulated genes in PMN-MDSC vs. APC and I-MDSC vs. APC were selected for overall survival (OS) and disease-free survival (DFS) analyses using the GEPIA2 database, on a cohort of 269 patients with colon adenocarcinoma (COAD) from the Cancer Genome Atlas (TCGA) dataset. The 200 genes were subjected to univariate survival analysis and genes exhibiting poor survival and significant Log-rank test *P* value (≤0.05) were identified. The refined gene list was then subjected to forward combined gene survival analysis and genes that improved the performance (lower Log-rank test *P* value) were retained, while those that did not were dropped from the signature. Analysis were conducted employing the GEPIA2 algorithm, as detailed previously (24). Patients were divided into high and low groups based on median gene expression; top 50% was designated as high and bottom 50% was designated as low, and the Log-rank test was used for curve comparison. The survival signature score is calculated by mean value of log_2_(TPM + 1) of each gene.

### Statistical Analyses

Statistical analyses were performed using GraphPad Prism 8 software (GraphPad Software, California, USA). For samples that passed the Shapiro-Wilk normality test, paired t-tests were performed, while those which did not pass the normality test were subjected to Wilcoxon signed-rank tests. Statistically significant *P* values are represented as follows; ***P < 0.001, **P < 0.01, *P < 0.05.

## Results

### Hierarchical Clustering and Comparisons of Differentially Expressed Genes in Colorectal Tumor-Infiltrating PMN-MDSC, I-MDSC-MDSC, and APCs

We have previously reported that levels of PMN-MDSCs, I-MDSCs, and APCs in TT are higher than those in NT from the same CRC patients (7, 14). In this study, we performed RNA-Seq to characterize the differential gene expression in these myeloid subsets. The gating strategy for sorting myeloid subsets was previously described (14) and as shown in **Figure 1**. CD33^+^HLA-DR^-/low^CD14^-^CD15^+^ were identified as PMN-MDSCs, CD33^+^HLA-DR^-/low^CD14^-^CD15^-^ were identified as I-MDSCs, and CD33^+^HLA-DR^+^CD14^+^ were identified as APCs.

**Figure 2:**
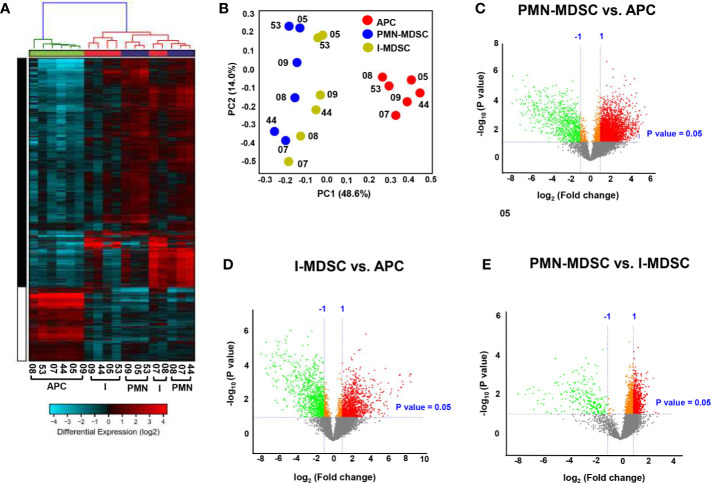
Hierarchical clustering and comparison of myeloid cell subpopulations (PMN-MDSCs, I-MDSCs and APCs) in tumor tissue of CRC patients. Cells isolated from tumor tissues of six CRC patients were stained for myeloid cell markers and sorted for RNA extraction. Hierarchical clustering of APCs, I-MDSCs and PMN-MDSCs from six tumor tissues (CRC patients #05, 07, 08, 09, 44 and 53) based on differentially-expressed RNA transcripts. Each column represents a sample and each row represents a transcript. Expression level of each gene in a single sample is depicted according to color scale **(A)**. Principle component analysis (PCA) based on the differentially expressed genes in each myeloid subpopulation **(B)**. Volcano plots show genes that were upregulated (shown in red), downregulated (shown in green) or remained unchanged (shown in grey) when comparing PMN-MDSC vs. APC **(C)**, I-MDSC vs. APC **(D)** and PMN-MDSC vs. I-MDSC **(E)**.

The hierarchal clustering shows a distinct cluster of genes which are differentially regulated in the tumor-infiltrating PMN-MDSC and I-MDSC, compared with APCs, from six CRC patients (**Figure 2A**). We found that a total of 3,133 upregulated genes in PMN-MDSC and 824 downregulated genes, compared to APCs. A total of 1,206 genes were upregulated in I-MDSC and 940 genes were downregulated in I-MDSC, compared to APCs. Additionally, we found that 788 genes were upregulated and 253 genes were downregulated in PMN-MDSC, compared to I-MDSC. Principal component analysis (PCA) showed that datasets for the myeloid populations (PMN/I-MDSC) were clustered distinctly from the control APCs suggesting the similarity of gene expression patterns in MDSC subsets, compared to APCs (**Figure 2B**). MDSCs clustered distinctly from APCs within the first two principal components, accounting for approximately 48.6% of the observed variation (**Figure 2B**). Volcano plot shows the genes that were upregulated (shown in red), downregulated (shown in green), or remained unchanged (shown in grey) when comparing PMN-MDSC vs. APC (**Figure 2C**), I-MDSC vs. APC (**Figure 2D**) and PMN-MDSC vs. I-MDSC (**Figure 2E**). Only genes that were significantly up/down-regulated, with a fold change > 2 and *P* value < 0.05 cutoffs were selected for subsequent analyses.

### Functional Annotation Analyses of Colorectal Tumor-Infiltrating PMN-MDSCs and I-MDSC-MDSCs

**Figure 3:**
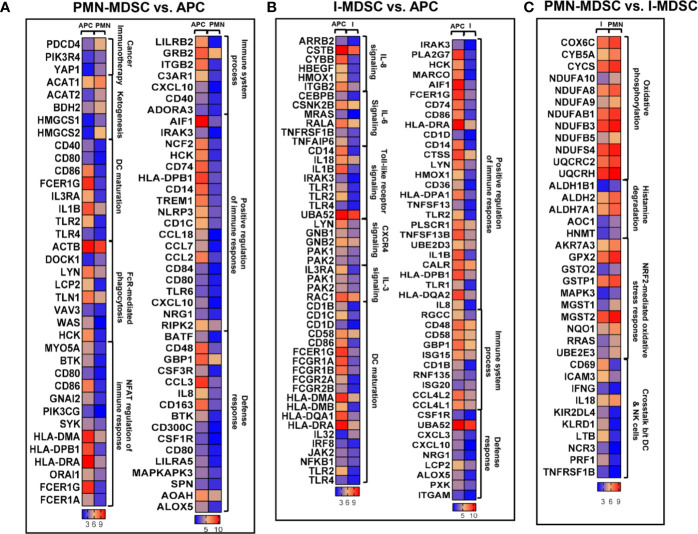
Differential gene expression of PMN-MDSCs, I-MDSCs and APCs in CRC tumor microenvironment. Functional categorization of top significantly upregulated and downregulated transcripts (with a fold change > 2, *P* value cutoff < 0.05) from CLC analysis were analyzed through IPA. Heat maps show the TPM representing fold change relative to the mean expression in PMN-MDSC (denoted as PMN) vs. APC **(A)**, I-MDSC (denoted as I) vs. APC **(B)** and PMN-MDSC vs. I-MDSC **(C)**.

APCs are responsible for antigen processing and presentation to activate adaptive immune responses, while MDSCs are known to suppress immune responses (25). In agreement with this, our RNA-Seq data confirmed these functional characteristics of myeloid subpopulations. We analyzed the differentially expressed genes in colorectal tumor-infiltrating PMN-MDSCs and I-MDSCs, compared to APCs (**Figures 3A, B**). Functional annotation analyses for top significantly affected transcripts, with a fold change > 2 and *P* value < 0.05 cutoffs, showed that genes related to immune system processes, positive regulation of immune response, and defense responses were downregulated in PMN-MDSCs, compared to APCs (**Figure 3A**). Additionally, genes involved in DC maturation, Fcγ receptor-mediated phagocytosis and NFAT-mediated regulation of immune response were significantly downregulated in PMN-MDSCs (**Figure 2A**). On the other hand, genes related to ketogenesis and potentially associated with resistance to cancer immunotherapy (anti-PD-1/PD-L1) were upregulated in PMN-MDSCs (**Figure 3A**). We also found that genes involved in the positive regulation of immune response, immune system processes and defense responses were downregulated in I-MDSCs (**Figure 2B**). Furthermore, genes related to IL-8, IL-6, CXCR4 and IL-3 signaling pathways, Toll-like receptor signaling pathways and DC maturation were significantly downregulated in I-MDSCs (**Figure 3B**). These data showed the functional characteristics of PMN-MDSCs and I-MDSCs as compared to APCs, and the different pathways associated with immune response regulation, which were downregulated in tumor-infiltrating PMN-MDSCs and I-MDSCs from CRC patients.

We found that genes related to oxidative phosphorylation, histamine degradation and NF‐E2‐related factor 2 (NRF2)-mediated oxidative stress response were upregulated in PMN-MDSCs, compared to I-MDSCs (**Figure 3C**). However, genes associated with the crosstalk between DCs and natural killer (NK) cells were significantly downregulated in PMN-MDSCs, compared to I-MDSCs (**Figure 3C**). These findings suggest that pathways related to metabolism, stress response and interaction between DCs and NK cells vary in colorectal tumor-infiltrating I-MDSCs vs. PMN-MDSCs.

Next, we performed protein-protein interaction (PPI) and enrichment network analysis, using STRING web-based tool, to show the interactions of proteins within the significantly affected pathways within myeloid subsets. For this analysis, we selected deregulated genes from PMN-MDSC vs. APC (**Supplementary Figure 1**), I-MDSC vs. APC (**Supplementary Figure 2**) and PMN-MDSC vs. I-MDSC (**Supplementary Figure 3**). For PMN-MDSC vs. APC, STRING database identified 72 nodes and 120 edges with PPI enrichment *P* value <1.0E-16, average clustering coefficient of 0.404 and average node degree of 3.33 (**Supplementary Figure 1**). Additionally, for I-MDSC vs. APC STRING database identified 76 nodes and 191 edges with PPI enrichment *P* value <1.0E-16, average clustering coefficient of 0.493 and average node degree of 5.03 (**Supplementary Figure 2**). Finally, for PMN-MDSC vs. I-MDSC, STRING database identified 37 nodes and 74 edges with PPI enrichment *P* value <1.0E-16, average clustering coefficient of 0.706 and average node degree of 4 (**Supplementary Figure 3**). In concordance with the IPA pathway analyses, we identified significant network of immune regulation in both PMN-MDCS/I-MDSC vs. APC and oxidative phosphorylation in PMN-MDSC vs. I-MDSC (**Supplementary Figures 1**-**3**). These data further confirm the significance of identified networks form IPA pathway analyses.

### Up/Downregulated Canonical Pathways in Colorectal Tumor-Infiltrating PMN-MDSCs

**Figure 4:**
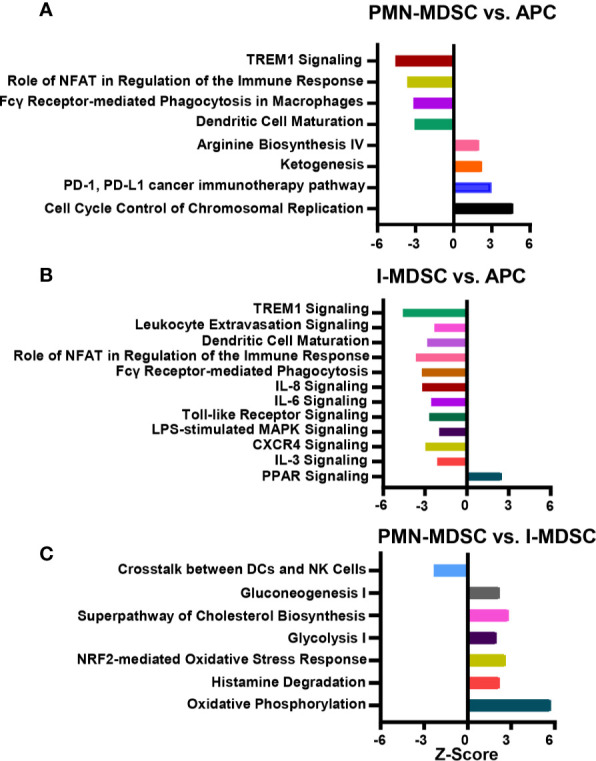
Canonical and signaling pathway analyses of myeloid subpopulations in the CRC tumor microenvironment. Functional categorization of top significantly upregulated and downregulated transcripts (with a fold change > 2, *P* value cutoff < 0.05) from CLC analysis were analyzed through IPA. Top significantly affected (1.5 > Z score < -2.0) canonical pathways were analyzed by IPA. The horizontal bars denote the different pathways based on the Z-scores; PMN-MDSC vs. APC **(A)**, I-MDSC vs. APC **(B)** and PMN-MDSC vs. I-MDSC **(C)**.

Next, differentially expressed genes in colorectal tumor-infiltrating myeloid subpopulations were subjected to Ingenuity Pathway Analysis (IPA). Based on our analysis, we identified canonical and signaling pathways that were differentially upregulated or downregulated in tumor-infiltrating PMN-MDSCs and I-MDSCs (**Figure 4**). We found that TREM1 signaling, NFAT regulation of the immune response, Fcγ receptor-mediated phagocytosis in macrophages and monocytes and DC maturation were downregulated in PMN-MDSCs, compared to APCs (-5.0 < Z score > -3.0, **Figure 4A**). On the other hand, pathways related to arginine biosynthesis, cancer immunotherapy and cell cycle were upregulated in PMN-MDSCs (2.0 < Z score > 3.0, **Figure 4A**).

### Up/Downregulated Canonical Pathways in Colorectal Tumor-Infiltrating I-MDSCs

We found that pathways related to TREM1 signaling, leukocyte extravasation, DC maturation, NFAT regulation of the immune response, Fcγ receptor-mediated phagocytosis in macrophages and monocytes, IL-8 signaling, IL-6 signaling, Toll-like receptor signaling, LPS-mediated MAPK signaling, CXCR4 and IL-3 signaling were all downregulated in I-MDSCs, compared to APCs (-4.0 < Z score > -2.0, **Figure 4B**). On the other hand, PPAR pathway was upregulated in I-MDSCs (**Figure 4B**).

### Up/Downregulated Canonical Pathways in Colorectal Tumor-Infiltrating PMN-MDSCs

Next, we compared pathways that were differentially upregulated or downregulated in PMN-MDSCs, compared to I-MDSCs. We found that pathways related to the crosstalk between DCs and NK cells were downregulated, while pathways related to oxidative phosphorylation, ketogenesis, glycolysis, glucogenesis, cholesterol biosynthesis and NRF2-mediated oxidative stress response were all upregulated in PMN-MDSC, compared with I-MDSCs (-3.0 < Z score > 6.0, **Figure 4C**). These findings suggest that pathways related to metabolism, stress response, histamine degradation and interaction between DCs and NK cells are different in tumor-infiltrating I-MDSCs and PMN-MDSCs. Thus, they provide novel insights into the functional pathways regulated by each MDSC subset in the colorectal TME.

### qRT-PCR Validation of Selected Gene Expression in Colorectal Tumor-Infiltrating PMN-MDSC and I-MDSC

**Figure 5:**
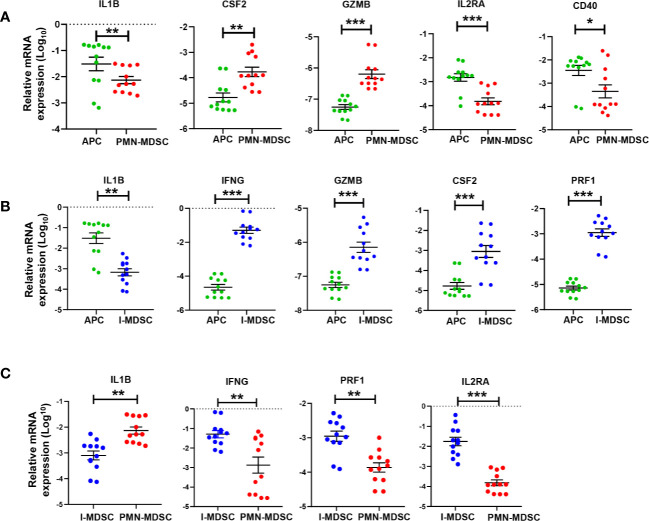
qRT-PCR validation of selected genes in tumor-infiltrating APCs, PMN-MDSCs and I-MDSCs from CRC tumor tissue. mRNA expression levels for selected genes in sorted tumor-infiltrating myeloid cells were validated by RT-PCR; PMN-MDSC vs. APC **(A)**; I-MDSC vs. APC **(B)** and PMN-MDSC vs. I-MDSC **(C)**. The relative gene expression was normalized to β-actin. Results obtained from two technical replicates of six CRC patients. The *P* values are indicated as follows; ***P < 0.001, **P < 0.01, *P < 0.05. Data are presented as the mean (Log_10_) ± standard error of the mean (SEM).

We validated the expression of selected genes from RNA-Seq data in tumor-infiltrating PMN-MDSCs, I-MDSCs and APCs (the latter used as a control) by qRT-PCR. The selected genes were amongst the top significantly affected transcripts with a fold of change >2 and *P* value cutoff <0.05. We validated that IL1B, IL2RA, and CD40 genes were downregulated in PMN-MDSCs (**Figure 5A**); these genes are important for APC and T cell activation (26–28). We validated that CSF2 and GZMB gene expressions were upregulated in PMN-MDSC (**Figure 5A**); these genes encode GM-CSF and granzyme B, respectively. Upregulated expression of GM-CSF and granzyme B in PMN-MDSC could be associated with their capability of recruiting neutrophils in the TME, which potentially increases tumor-associated neutrophils, and releasing cytolytic molecules to kill immune cells that positively regulate anti-tumor immunity (29). We also validated the downregulation of IL1B and the upregulation of IFNG, GZMB, CSF2, and PRF1 genes in I-MDSCs, compared to APCs (**Figure 5B**). Although the upregulated genes, IFNG, GZMB and PRF1 (PRF1 encodes perforin 1), have been implicated in CD8^+^ T cell activation and function (30), they could be also associated with the promotion of I-MDSC suppressive function to enhance PD-L1 expression and kill reactive CD8^+^ T cells and favor tumor growth (31–33). Finally, we validated the upregulation of IL1B and the downregulation of IFNG and IL2RA genes in PMN-MDSCs, compared to I-MDSCs (**Figure 5C**). This latter finding suggests that PMN-MDSCs and I-MDSCs can mediate different pathways and induce different effects on the immune response and tumor microenvironment.

### TCGA Analysis Revealed Gene Signatures in Tumor-Infiltrating PMN-MDSC and I-MDSC Associated With Poor Prognosis in Patients With Colorectal Adenocarcinoma

**Figure 6:**
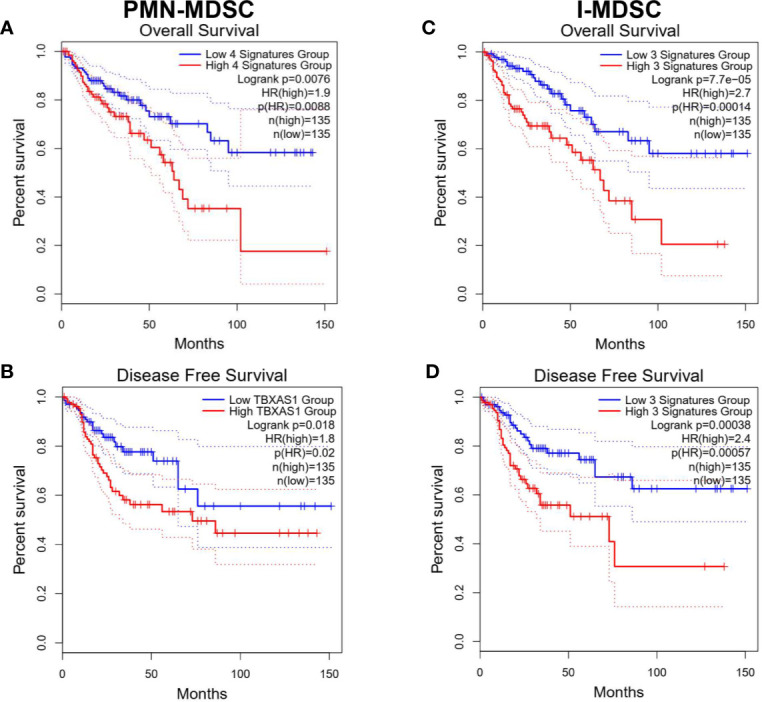
Kaplan-Meier curves for overall survival and disease-free survival according to the identified gene signatures in tumor-infiltrating PMN-MDSCs and I-MDSCs. Patients were divided into high and low groups based on median gene expression. Kaplan-Meier curves illustrate the duration of overall survival (OS) **(A**, **C)** and disease-free survival (DFS) of 270 patients **(B**, **D)** according to the expression of identified gene signatures in cohorts of patients in the Cancer Genome Atlas (TCGA) colorectal adenocarcinoma dataset. The Log-rank test was used for curve comparison. The survival signature score is calculated by mean value of log_2_(TPM7nbsp;+ 1) of each gene.

Survival analysis of top downregulated genes in PMN-MDSC vs. APC identified NPL, CATSPER1, PRAM1, SLC11A1, APOE, and TREM2 as poor OS prognostic markers in COAD using univariate analysis. High gene signature of TREM2, CATSPER1, NPL, and PRAM1 had the highest prognostic value (Log-rank *P* = 0.0076, HR(high)=1.9) (**Figure 6A**). On the other hand, high signature of RBP7, IL3RA, and TBXAS1 correlated with worse DFS in COAD. TBXAS1 alone exhibited the highest prognostic value [Log-rank *P* = 0.018, HR(high)=1.8] for DFS (**Figure 6B**). Additionally, I-MDSC vs. APC-derived gene signature was subjected to OS and DFS analysis. Using univariate analysis, we found that GPNMB, INHBA, CATSPER1, HAMP, HTRA4, NPL, PRAM1, TNNT1, SLC11A1, and TREM2 predicted worse OS, while INHBA, TNNT1, and IL3RA predicted worse DFS. Combination of TNNT1, NPL, and CATSPER1 had the strongest prognostic power for OS [Log-rank *P* = 7.7e−05, HR(high)=2.7], while combination of (TNNT1, IL3RA, and INHBA) was associated with worst DFS [Log-rank *P* = 0.00038, HR(high)=2.4] (**Figures 6C, D**). It is worth noting that top upregulated genes in PMN-MDSC or I-MDSC vs. APC had little impact on prognosis and survival rates, hence, they were not included in these analyses.

## Discussion

Increased MDSC numbers in CRC tumor tissues, compared to paired-normal tissues have been previously reported, implicating the importance of MDSC function in tumorigenesis and immunosuppression (14). We have previously reported the transcriptomic profiles of CRC tumor-infiltrating I-MDSCs and PMN-MDSCs, compared with APCs (14). We found that pathways related to JNK and Wnt signaling, and SNARE complex activation were upregulated in I-MDSCs. Meanwhile, pathways related to CRC progression, cell migration, and MDSC recruitment were upregulated in PMN-MDSCs (14). These findings were obtained from RNA-Seq data analyses in two CRC patients (14). In this study, we included more patient samples in order to identify additional variations and emergence of different signaling pathways. Moreover, it is noteworthy that sorting few cells of different myeloid subsets; therefore, it was challenging to perform RNA extraction from few cells and additional amplification steps were required. Few studies have reported that cryopreservation/thawing procedure can reduce the proportion of MDSC subsets in the circulation (peripheral blood mononuclear cells; PBMC) and the expression of arginase-1 (34, 35). However, the use of cryopreserved PBMC samples is acceptable as no significant changes were seen in the number of the most suppressive MDSC subset, PMN-MDSC, in fresh versus cryopreserved PBMC (36). We have also shown that expression of arginase-1 mRNA in CD33^+^HLA-DR^-^ myeloid cells, presumably suppressive subsets of myeloid cells, in cryopreserved PBMC is higher than that of CD33^+^HLA-DR^+^ APCs (37). Additionally, we have sorted different MDSC subsets from tumor tissue, which could be less susceptible to be affected by freeze-thaw procedure, unlike PBMC.

Hierarchical cluster analysis and PCA showed that variations within myeloid subsets (I-MDSCs and PMN-MDSCs) are much less than those compared between myeloid subsets and APCs. These data confirm the differences in the functional characteristics of MDSCs versus APCs. In support of this, we found that genes associated with DC maturation, positive regulation of immune response, immune system processes and defense response were downregulated in PMN-MDSCs, compared with APCs. DCs, which also function as APCs, play an important role in the activation of anti-tumor immunity (15). DC maturation is very important for the activation of adaptive immunity and T cell responses (15). One of the mechanisms by which cancer cells evade anti-tumor immunity is by compromising APC function and DC maturation (38). Genetic, epigenetic and cell-mediated mechanisms can contribute to APC dysfunction and impaired DC maturation (39). Some of these cell-mediated mechanisms are driven by the action of MDSCs. Tan et al. reported that PMN-MDSCs, induced by modified vaccinia TianTan in mesothelioma mouse model, suppress DC function by releasing IL-10, resulting in the impaired induction of anti-tumor cytotoxic T cell response (40). In another study, PMN-MDSCs were shown to suppress DC maturation and T cell proliferation in autoimmune arthritis mouse model (41). In addition to DC maturation, we found that other immune response-related pathways, such as TREM1 signaling (16), NFAT-mediated regulation of immune response (17) and Fcγ receptor-mediated phagocytosis (18), were downregulated in PMN-MDSCs, compared to APCs. On the other hand, PMN-MDSCs showed an upregulation of pathways related to arginine biosynthesis, which potentially lead to increased production of arginine and its consumption by tumor cells to maintain their survival and growth (42, 43), and pathways potentially associated with resistance to cancer immunotherapy primarily anti-PD-1/anti-PD-L1 therapy (44).

In contrast to tumor-infiltrating APCs, pathways related to the positive regulation of immune and defense response, including IL-8/IL-6/IL-3/CXCR4 signaling, TREM1 signaling and leukocyte extravasation pathways were downregulated in I-MDSCs. Several cytokines signaling pathways, such as IL-8, IL-6, and IL-3 have been implicated in the positive regulation of immune response and the activation of T cell responses. IL-8 (45) and IL-6 (46) signaling pathways have a double-sword function in anti-tumor immunity. IL-8 signaling is known to recruit leukocytes to the TME *via* a chemoattractant gradient; these leukocytes play a role in innate immunity and could promote anti-tumor immune responses (45). It has been demonstrated that IL-6 production by TLR-activated APCs drives the activation of CD4^+^ T cells and B cell antibody response, suggesting a role for IL-6 signaling in the activation of immune responses (47). NFAT-mediated regulation of immune response and DC maturation pathways were found to be downregulated in both PMN- and I-MDSCs. NFAT is a key regulator for several key pathways, including IL-3 production, which is responsible for both lymphoid and myeloid differentiation (48). We found that IL-3RA gene was downregulated in both PMN- and I-MDSCs, compared with APCs (**Figures 2A, B**). Together, these data suggest the potential relationship between NFAT and IL-3 pathways, and may indicate that downregulation of NFAT-IL-3 pathways interfere with the maturation of myeloid cells in the CRC TME.

We also compared the transcriptional profiles of CRC tumor-infiltrating PMN-MDSCs with I-MDSCs. RNA-Seq data, canonical pathway and IPA analyses showed that genes and pathways related to the crosstalk between DCs and NK cells were downregulated in PMN-MDSCs, while metabolic pathways related to oxidative phosphorylation, ketogenesis, glycolysis, glucogenesis, cholesterol biosynthesis and NRF2-mediated oxidative stress response were all upregulated in PMN-MDSC. These pathways could lead to the increased consumption of glucose, cholesterol metabolism, reduced mitochondrial respiration, and increased generation of reactive oxygen species, which all promote tumor growth and survival (49). PMN-MDSCs could be responsible for enhancing the immunosuppressive environment within the tumor by mediating glycolysis and oxidative phosphorylation, leading to lipid accumulation (50). In turn, lipid uptake by tumor-infiltrating MDSCs enhances their immunosuppressive activities, which they exert on T cells, thereby promoting tumorigenesis (50). These results may reflect the phenotypical, molecular and functional characteristics of each MDSC subset in the CRC TME.

Based on TCGA analysis for the downregulated genes from our RNA-Seq data, high gene signature comprising of TREM2, CATSPER1, NPL, and PRAM1 in tumor-infiltrating PMN-MDSC predicted poor OS rates in COAD patients. Triggering receptor expressed on myeloid cells 2 (TREM2) gene is important for the activation of macrophages and DCs, and secretion of inflammatory cytokines, while PML-RARA Regulated Adaptor Molecule 1 (PRAM1) gene could be associated with neutrophil function, myeloid differentiation and T cell activation (51–54). Therefore, their downregulation in PMN-MDSC could negatively influence anti-tumor immune responses and favor disease progression. However, the function of CATSPER1 and NPL in myeloid cell biology and function remains unclear. Additionally, high gene signature, with only thromboxane A synthase 1 (TBXAS1), in PMN-MDSC was found to be the best predictor for short DFS in patients. The role of TBXAS1 has been implicated in DC maturation and Th response, inflammatory response in myeloid cells and lipid metabolism (50, 55). Hence, low levels of TBXAS1 could favor tumor escape from anti-tumor immunity and may also support PMN-MDSC suppressive functions. In tumor-infiltrating I-MDSC, high gene signature comprising of TNNT1, NPL and CATSPER1 predicted poor OS rates in COAD patients. Moreover, high signature of TNNT1, IL3RA and INHBA in I-MDSC predicted short DFS in COAD patients. Downregulation of IL-3 signaling pathway is associated with impairment of DC maturation and activation of adaptive immune response (56), and hence could be associated with diminished ability of tumor eradication, and poor survival rates. Like NPL and CATSPER1, the roles of TNNT1, and INHBA (gene encodes a member of TGF-β family and act as a growth/differentiation factor) in myeloid cell biology and function are not clear.

Together, our findings highlight different molecular and functional pathways regulated by PMN-MDSCs and I-MDSCs in CRC TME, compared to APCs, and provide novel insights into gene signatures for each subset, which could predict poor OS and DFS in CRC patients. Some of these genes/pathways could be targeted in MDSCs and could have a clinical benefit in CRC patients. Notably, this study included six CRC patients and there were no functional assays used to validate the findings presented in the manuscript.

## Data Availability Statement

The datasets presented in this study can be found in online repositories. The names of the repository/repositories and accession number(s) can be found below: BioProject ID PRJNA664006.

## Ethics Statement

The studies involving human participants were reviewed and approved by Hamad Medical Corporation, Doha, Qatar (protocol no. MRC-02-18-012) and Qatar Biomedical Research Institute, Doha, Qatar (protocol no. 2018-018). The patients/participants provided their written informed consent to participate in this study.

## Author Contributions

RS: Data curation, Methodology, Formal analysis, Investigation, Writing the original draft. VS: Data curation, Methodology, Formal analysis, Investigation, Writing-review and editing. MA-D, MK, and MA: Sample acquisition, Investigation. NA: Formal analysis, Methodology, Writing-review and editing. EE: Conceptualization, Resources, Data curation, Software, Formal analysis, Supervision, Funding acquisition, Validation, Investigation, Visualization, Methodology, Project administration, Writing-review and editing. All authors contributed to the article and approved the submitted version.

## Funding

This work was supported by a start-up grant [VR04] for EE from Qatar Biomedical Research Institute, Qatar Foundation.

## Conflict of Interest

The authors declare that the research was conducted in the absence of any commercial or financial relationships that could be construed as a potential conflict of interest.

## Supplementary Material

Supplementary Figure 1 Protein-protein interaction networks for the significantly affected pathways in PMN-MDSC vs. APC. PPI networks for top significantly upregulated and downregulated transcripts (with a fold change > 2, *P* value cutoff < 0.05) pathways in PMN-MDSC vs. APC were obtained from STRING database as described in methods. The significance of the network and the code for Gene ontology (GO) terms (Biological Process) and False discovery rate (FDR) for each term are shown in the table.

Supplementary Figure 2 Protein-protein interaction networks for the significantly affected pathways in I-MDSC vs. APC. PPI networks for top significantly upregulated and downregulated transcripts (with a fold change > 2, *P* value cutoff < 0.05) pathways in I-MDSC vs. APC were obtained from STRING database as described in methods. The significance of the network and the code for Gene ontology (GO) terms (Biological Process) and False discovery rate (FDR) for each term are shown in the table.

Supplementary Figure 3 Protein-protein interaction networks for the significantly affected pathways in PMN-MDSC vs. I-MDSC. PPI networks for top significantly upregulated and downregulated transcripts (with a fold change > 2, *P* value cutoff < 0.05) pathways in PMN-MDSC vs. I-MDSC were obtained from STRING database as described in methods. The significance of the network and the code for Gene ontology (GO) terms (Biological Process) and False discovery rate (FDR) for each term are shown in the table.

## Abbreviations

APCs, Antigen presenting cells; CRC, Colorectal cancer; DC, Dendritic Cells; I-MDSCs, Immature-myeloid derived suppressor cells; IL-3, Interleukin-3; IL-6, Interleukin-6; MDSCs, Myeloid-derived suppressor cells; MAPK, Mitogen-activated protein kinase; NFAT, Nuclear factor of activated T-cells; PMN-MDSCs, polymorphonuclear/granulocytic-myeloid derived suppressor cells; PPI network, Protein-protein interaction network; TME, Tumor microenvironment.
