# Supplementary figures and images for: RNA-Seq Analysis of Colorectal Tumor-Infiltrating Myeloid-Derived Suppressor Cell Subsets Revealed Gene Signatures of Poor Prognosis
Source: Front Oncol. 2020 Nov 10;10:604906. doi: 10.3389/fonc.2020.604906 (PMC7703275; doi:10.3389/fonc.2020.604906)

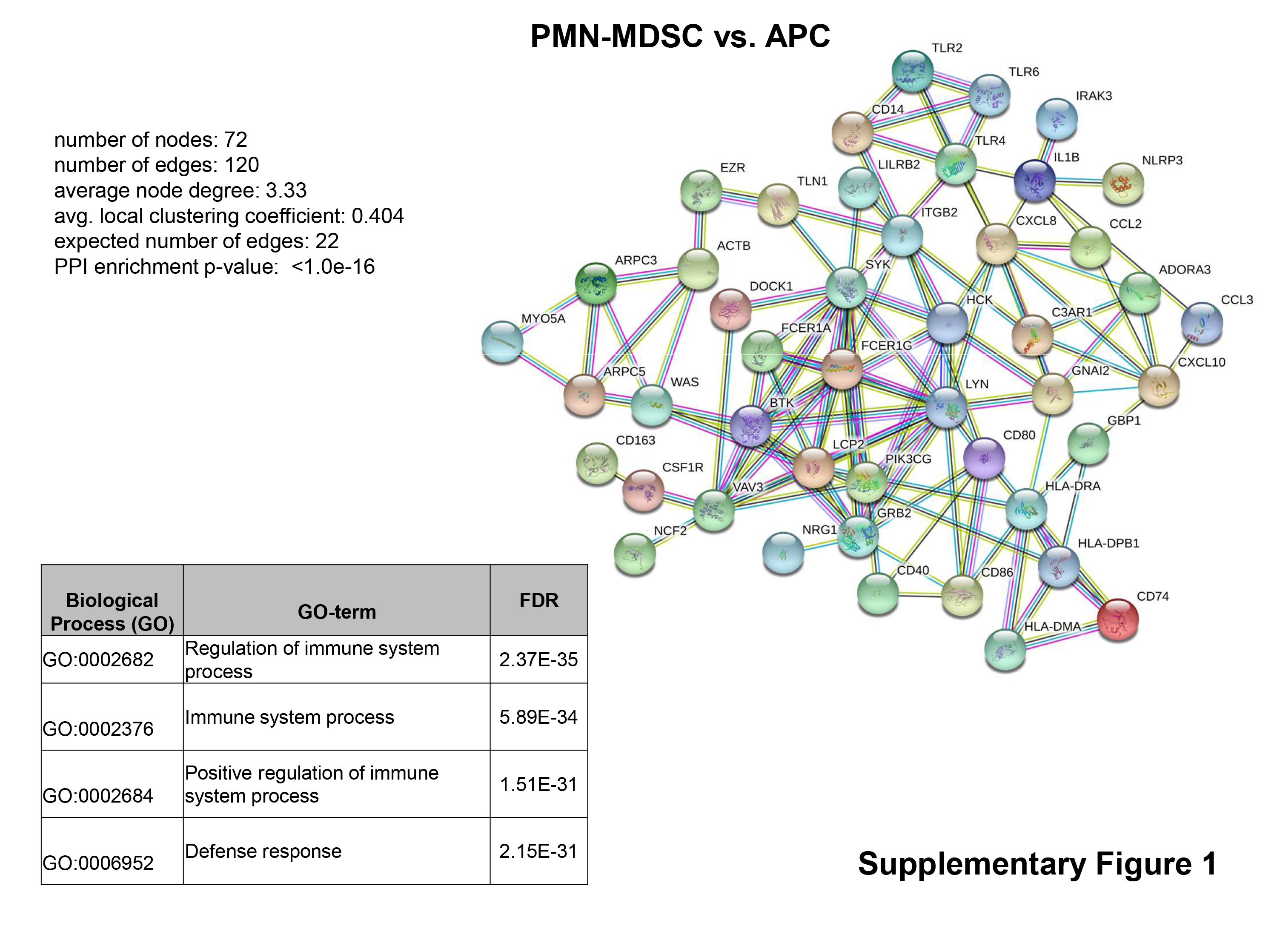

Supplement: Supplementary Figure 1 — Protein-protein interaction networks for the significantly affected pathways in PMN-MDSC vs. APC. PPI networks for top significantly upregulated and downregulated transcripts (with a fold change > 2, P value cutoff < 0.05) pathways in PMN-MDSC vs. APC were obtained from STRING database as described in methods. The significance of the network and the code for Gene ontology (GO) terms (Biological Process) and False discovery rate (FDR) for each term are shown in the table. [file Image_1.tif]

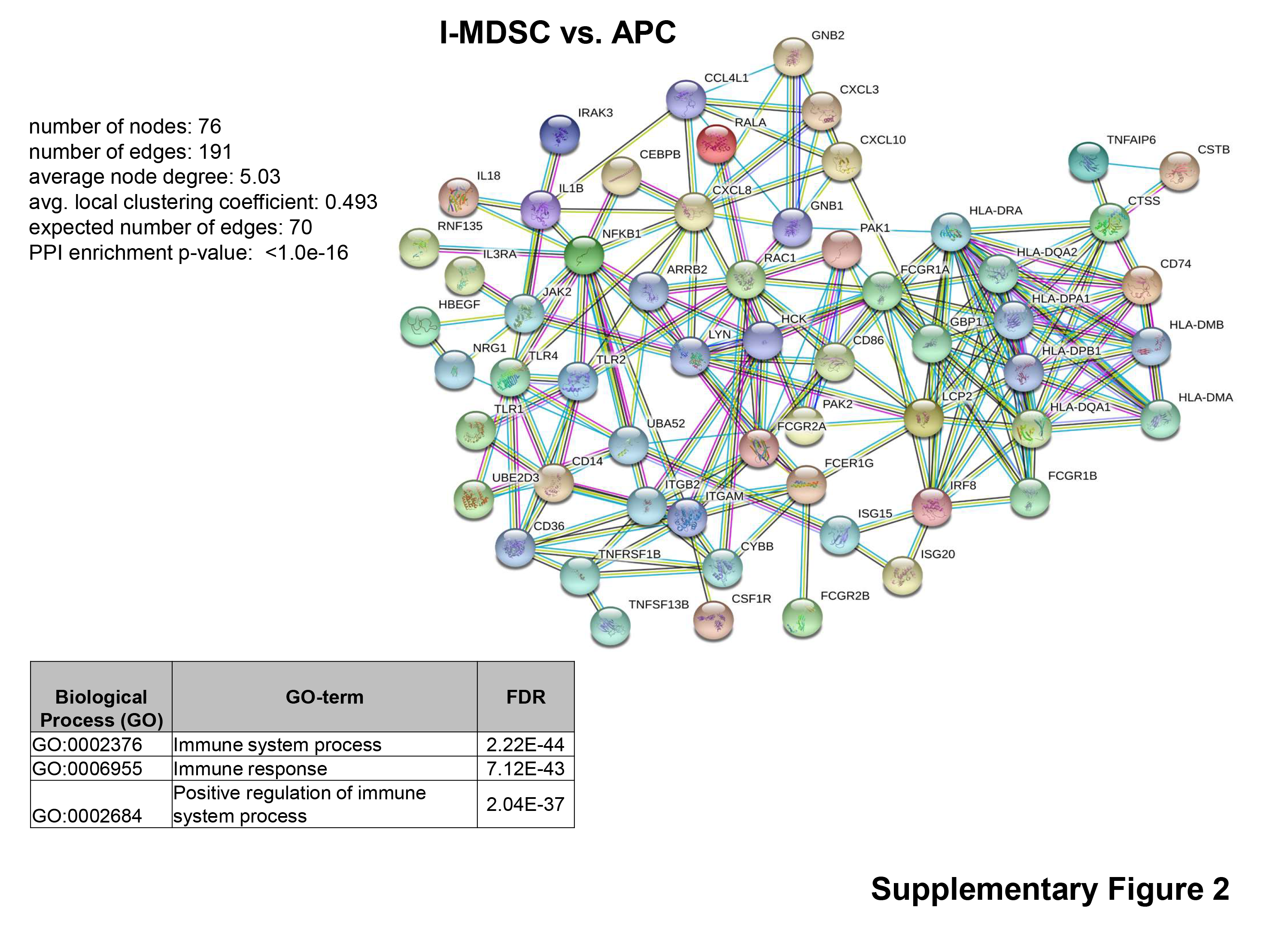

Supplement: Supplementary Figure 2 — Protein-protein interaction networks for the significantly affected pathways in I-MDSC vs. APC. PPI networks for top significantly upregulated and downregulated transcripts (with a fold change > 2, P value cutoff < 0.05) pathways in I-MDSC vs. APC were obtained from STRING database as described in methods. The significance of the network and the code for Gene ontology (GO) terms (Biological Process) and False discovery rate (FDR) for each term are shown in the table. [file Image_2.tif]

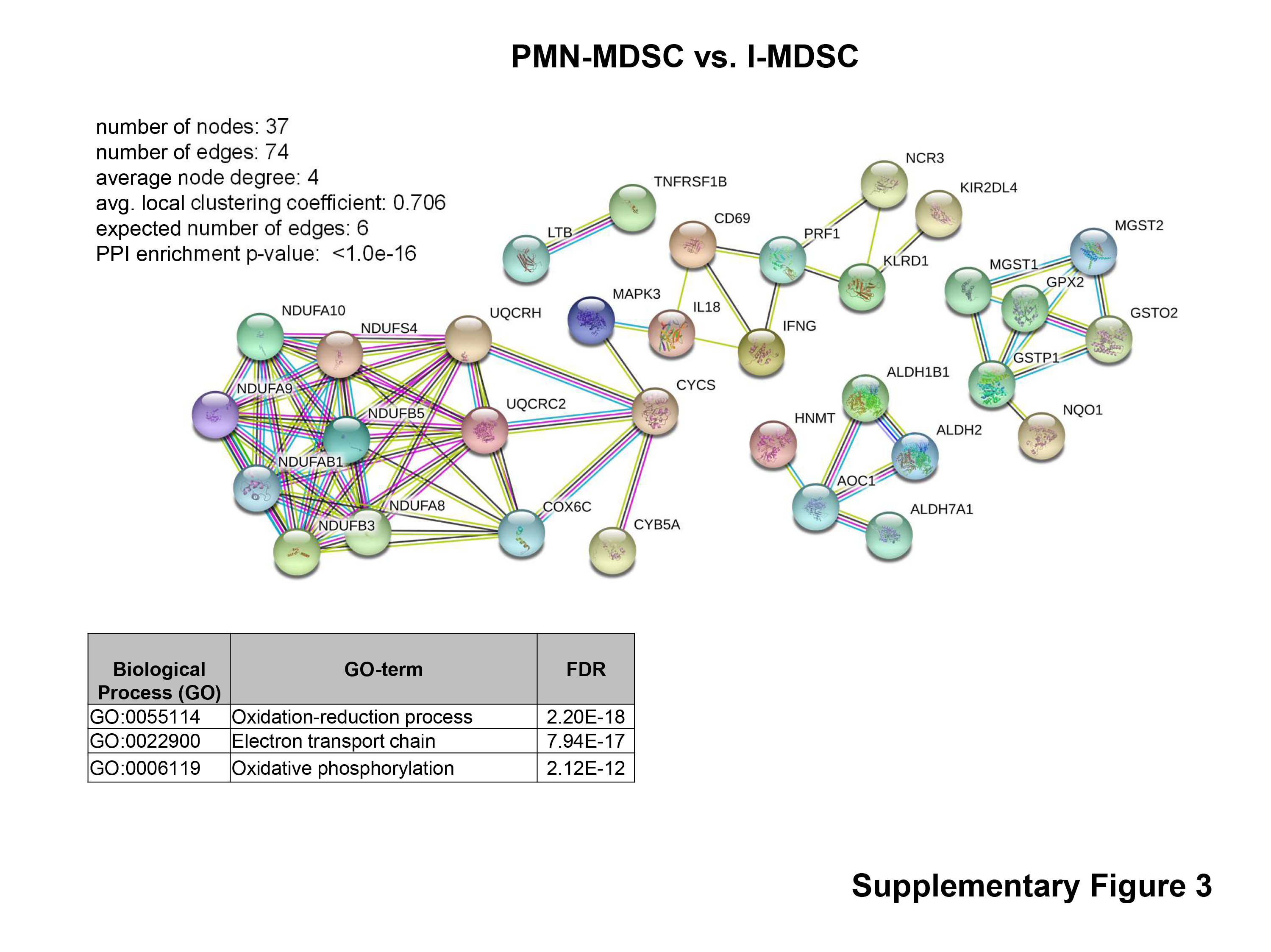

Supplement: Supplementary Figure 3 — Protein-protein interaction networks for the significantly affected pathways in PMN-MDSC vs. I-MDSC. PPI networks for top significantly upregulated and downregulated transcripts (with a fold change > 2, P value cutoff < 0.05) pathways in PMN-MDSC vs. I-MDSC were obtained from STRING database as described in methods. The significance of the network and the code for Gene ontology (GO) terms (Biological Process) and False discovery rate (FDR) for each term are shown in the table. [file Image_3.tif]
