# Supplementary material for: RNA-Seq Analysis of Colorectal Tumor-Infiltrating Myeloid-Derived Suppressor Cell Subsets Revealed Gene Signatures of Poor Prognosis
Source: Front Oncol. 2020 Nov 10;10:604906. doi: 10.3389/fonc.2020.604906 (PMC7703275; doi:10.3389/fonc.2020.604906)
Supplement: Supplementary file 5 [file Table_2.docx]

**Supplementary Table 2. Primer sequences for the qRT-PCR.**

| **Primer** | **Sequence** |
| --- | --- |
| CD40 | Forward, 5’- ACTGAAACGGAATGCCTTCCT -3’  Reverse, 5’- CCTCACTCGTACAGTGCCA -3’ |
| CSF2 | Forward, 5’- TCCTGAACCTGAGTAGAGACAC -3’  Reverse, 5’- TGCTGCTTGTAGTGGCTGG-3’ |
| IL1B | Forward, 5’- ATGATGGCTTATTACAGTGGCAA-3’  Reverse, 5’- GTCGGAGATTCGTAGCTGGA -3’ |
| PRF1 | Forward, 5’- GCTGGACGTGACTCCTAAGC-3’  Reverse, 5’- GATGAAGTGGGTGCCGTAGT -3’ |
| GZMB | Forward, 5’- GTAAGGGGGAAACAACAGCA -3’  Reverse, 5’- CCCCAAGGTGACATTTATGG -3’ |
| IL2RA | Forward, 5’- GTGGGGACTGCTCACGTTC -3’  Reverse, 5’- CCCGCTTTTTATTCTGCGGAA -3’ |
| IFNG | Forward, 5’- TGACCAGAGCATCCAAAAGA -3’  Reverse, 5’- CTCTTCGACCTCGAAACAGC -3’ |
| β-ACTIN | Forward, 5’- AGAGCTACGAGCTGCCTGAC -3’  Reverse, 5’- AGCACTGTGTTGGCGTACAG -3’ |
